# A Novel Piggybac Transposon Inducible Expression System Identifies a Role for Akt Signalling in Primordial Germ Cell Migration

**DOI:** 10.1371/journal.pone.0077222

**Published:** 2013-11-04

**Authors:** James D. Glover, Lorna Taylor, Adrian Sherman, Caroline Zeiger-Poli, Helen M. Sang, Michael J. McGrew

**Affiliations:** The Roslin Institute and Royal (Dick) School of Veterinary Studies, University of Edinburgh, Easter Bush Campus, Midlothian, United Kingdom; Baylor College of Medicine, United States of America

## Abstract

In this work, we describe a single piggyBac transposon system containing both a tet-activator and a doxycycline-inducible expression cassette. We demonstrate that a gene product can be conditionally expressed from the integrated transposon and a second gene can be simultaneously targeted by a short hairpin RNA contained within the transposon, both *in vivo* and in mammalian and avian cell lines. We applied this system to stably modify chicken primordial germ cell (PGC) lines *in vitro* and induce a reporter gene at specific developmental stages after injection of the transposon-modified germ cells into chicken embryos. We used this vector to express a constitutively-active AKT molecule during PGC migration to the forming gonad. We found that PGC migration was retarded and cells could not colonise the forming gonad. Correct levels of AKT activation are thus essential for germ cell migration during early embryonic development.

## Introduction

Regulated control of gene expression in animal species has been achieved in many biological model organisms from mouse to *Drosophila* using inducible expression vectors [1], [2]. The tetracycline (tet)-regulated expression system consists of a vector carrying the tet responsive activator protein (TA) and a separate vector carrying a tet-responsive element (TRE) regulated promoter and the gene product to be expressed. Addition or withdrawal of the antibiotic tet, or its derivatives, can either activate (Tet-on) or inhibit (Tet-off) transgene expression depending on the modifications to the TA protein [3]. Regulated gene knockdown using short hairpin RNA molecules (shRNAs), expressed by the Tet-on system has been demonstrated *in vitro* and in transgenic animals [4]–[8] Regulated gene expression for gene silencing is often optimal to avoid deleterious effects of knocking down essential genes for cell survival and early embryonic development, and for expressing proteins involved in cell growth and survival [9]. Recently, both lentiviral and plasmid vector systems containing both the TA and Tet-on transgenes have been developed [10], [11].

The piggyBac DNA transposon vector has been shown to be a highly efficient, non-viral system for transgene insertion into both chicken and mammalian cells [12]–[16]. PiggyBac-based vectors containing inducible tet-responsive transgenes have also been used for regulated generation of induced pluripotent cells and to modify gene expression in cells and transgenic animals [17]–[21].

In this study, we present a novel piggyBac vector carrying both a constitutively-expressed TA and a Tet-on inducible transgene. The integrated vector conditionally expresses a gene product whilst concomitantly reducing the expression of a second gene targeted by a shRNA molecule. We show that a piggyBac vector carrying a Tet-on inducible transgene can be stably integrated into mouse ES cells and chicken cell lines. We demonstrate that expression of a reporter gene is tightly regulated and endogenous gene expression can be reduced *in vitro* and *in vivo* upon the addition of doxycycline (dox). We used this system to modify the AKT signalling pathway in migratory chicken primordial germ cells (PGCs). PGCs are the precursors of the germ cell lineage that differentiate into the gametes in the adult. Early migratory PGCs can be cultured *in vitro* [22], [23] and are efficiently genetically-modified using piggyBac vectors [16]. Transposition efficiencies of 10.5% were obtained using piggyBac transposons [16]. We show that transgene expression can be induced in piggyBac-modified PGCs at various developmental stages including adult testes and in embryos developed after fertilisation by sperm derived from the modified PGCs. We used the piggyBac vector to express conditionally a constitutively active AKT molecule in migratory PGCs. AKT, a serine/threonine kinase, is implicated in germ cell survival and migration. We find that AKT signalling is critical for germ cell migration to the forming gonad.

## Materials and Methods

### Vector constructs

The transactivator, rtTA3, was isolated from pSLIK-NEO-TGmiRLuc [10] using *Eco*RI and subcloned into PB-CGIP [16] digested with *Eco*RI to replace the GFP gene to produce PB-CTA-IP. A TRE-Tight promoter was isolated from pEN-TTMCS [10] using *Sal*I, cloned into *Xho*I to produce PB-CTA-IP-TRE. To construct PB Tet-On GFP, a TRE-Tight promoter driving GFP (TRE-GFP) was isolated from pEN-TTGmiRc2 using *Sal*I, and subcloned into the *Xho*I site of PB-CTA-IP. A shuttle vector was used to transfer shRNAs and cDNAs into PB-CTA-IP-TRE. A fragment containing shGFP surrounded by chicken miR flanking regions was isolated from pRFPRNAiGFP [24] using *Kpn*I/*Spe*I and cloned into pGEM-T Easy digested with *Mlu*I/*Nco*I. The rabbit β-globin polyA was subcloned 3′ of the shGFP using *Sal*I. An Apple fluorescent protein cDNA [25] was cloned 5′of the shGFP hairpin knockdown construct using *Eag*I. The Apple shGFP polyA fragment was excised with *Age*I/*Mlu*I and subcloned into PB-CTA-IP-TRE, downstream of the TRE promoter at a *Pac*I site, to generate PB Tet-On Apple shGFP. A myristolated AKT molecule [26] was cloned into the pGEM T-Easy vector containing the rabbit β-globin polyA. The AKT polyA fragment was cut with *Age*I/*Mlu*I and cloned into the *Pac*I site of PB-CTA-IP-TRE to produce PB Tet-On AKT. A piggyBac vector driving a cherry reporter gene was made by cloning monomeric Cherry [27] using *Eco*RI/*Bam*HI into the *Eco*RI site of PB-CGIP, replacing the GFP gene.

### Cell Culture

PGCs were cultured on a monolayer of irradiated STO cells in knockout Dulbecco's modified Eagle's medium (Life Technologies) containing 12.5% BRL conditioned medium, 7.5% foetal bovine serum (FBS) ES cell grade (PAA Laboratories), 2.5% chicken serum (Biosera), 1× EmbryoMax nucleosides (Millipore), 1× glutamax (Life Technologies), 1× NEAA (Life Technologies), 0.1 mM β-mercaptoethanol (Life Technologies), 0.2 mM sodium pyruvate (Life Technologies) and 1× penicillin/streptomycin (P/S) (Sigma). PGCs for injection into embryos for generation of hatched birds were cultured in a modified basal medium with reduced serum conditions.

GFP^+^ chick embryo fibroblasts (CEFs) were isolated from day 10 ‘Roslin Green’ chicken embryos [28]. Embryos were dissected and macerated using surgical scissors before being passed through a 50 ml syringe with fine gauge needle. The CEFs were cultured in DMEM containing 10% FBS (Biosera), 1× Glutamax (Life Technologies) and 1× P/S (Sigma). Mouse ES cells (mESCs, Bruce4) were cultured in DMEM with 1× Glutamax (Life Technologies), 10% FBS gold (PAA Laboratories), 0.1 mM β-mercaptoethanol (Life Technologies), 0.1 mM MEM non-essential amino acids (Life Technologies), 1% P/S (Sigma) supplemented with 1000 U/mL human LIF (Millipore).

### Cell transfection and induction

The piggyBac vectors were co-transfected with the piggyBac transposase Cyc43 or Hybase. [16], [29]. The original piggyBac vectors were obtained from the Wellcome Trust Sanger Institute (Hinxton, United Kingdom). DNA was transfected at a 2 μg:2 μg transposon:transposase ratio in all cell types. Approximately 100,000 PGCs were transfected, using DMRIE-C (Life Technologies) and Fugene-HD and Lipofectamine 2000 (Life Technologies) were used to transfect CEFs and mESCs, respectively. One week post transfection, cells were selected with puromycin (Sigma). For expression assays, cells were induced with doxycycline hyclate (Sigma) at a final concentration of 1 μg/ml.

### Western blot

PGCs were collected and resuspended in PBS containing a complete protease inhibitor cocktail tablet (Roche), disrupted with a handheld homogeniser, followed by 3×30s of sonication. 10 µg of protein extract was separated on a 12% NuPage Bi-Tris gel (Life Technologies) and semi dry-transferred to ECL membrane (Amersham). Membranes were blocked in 5% milk/Tris-Buffered Saline Tween-20 (TBST) and incubated overnight with primary antibodies; anti-rabbit phospho-AKT (Ser473) XP, 1∶1000 (Cell Signalling) or anti-mouse γ-tubulin, 1∶2500 (Sigma) in 5% BSA/TBST. After several TBST washes, blots were incubated with HRP conjugated secondary antibodies, anti-rabbit immunoglobulins (Dako) or anti-mouse immunoglobulins (Dako), in 5% milk/TBST for 1hr at room temperature followed by washing in TBST. Blots were detected using Novex ECL chemiluminescent substrate reagent kit (Life Technologies) and exposed to Hyperfilm ECL (Amersham).

### Embryo electroporation

A DNA solution of 1 μg/μl PB CAG Tet-On shGFP and 1 μg/μl PB CAG:Hybase diluted in PBS with fast green dye, was injected into the neural tube of stage 16HH [30] embryos in windowed eggs. Using microelectrodes, embryos were electroporated at 25 V, 50 ms intervals, 6 pulses. After electroporation, eggs were resealed and the embryos incubated for seven days. On day seven, eggs were injected with 1 ml of doxycycline (0.1 mg/ml dissolved in Hank's Balanced Salt Solution and then every 48 hrs for a further 7 days. At day 16 of incubation, embryos were isolated and the spinal cord was dissected, fixed in 4% PFA and embedded for cryosectioning.

### PGC transplantations into host embryos and semen screening

GFP^+^ PGCs stably modified with PB Tet-On Apple shGFP were injected into the dorsal aorta of stage HH16 embryos. Donor embryos were transferred into phase III host shells as previously described in [31]. Chicks were raised to sexual maturity and semen from founder males was screened for the apple transgene.

### PCR screening for the apple transgene and Southern blot analysis

Semen and offspring were screened for transgene presence through semi-quantitative PCR as described in [32]. Briefly, 50 ng of genomic DNA from semen or animal tissues was amplified using TA-specific primers (GAGAACGTATGTCGAGGTAGGCG, CTTCAGCTTAGCGGTCTGAA) or GFP transgene-specific primers (ACCAGTAGTTAATTTCTGAGACCCTTGTA, CGAGATCCTACAGTTGGCGCCCGAACAG) and compared to plasmid PCR controls. Two founder G_0_ males were crossed with wild type stock hens and GFP-expressing offspring identified (see McGrew et al. [33]). For Southern blot analysis 5 μg of genomic DNA isolated from G_1_ embryos or cells was digested overnight using the appropriate restriction enzymes. The DNA digests were resolved by gel electrophoresis and transferred to HybondN membrane (GE Healthcare). PB-Tet-On GFP vector was digested to isolate a 0.6 kb DNA fragment that was then labeled with [α-^32^P]dCTP using the DIG High Prime DNA Labeling and Detection Kit II (Roche) and used to probe the Southern blot.

All experiments involving animals, animal breeding or animal care procedures were approved by The Roslin Institute's animal ethics committee. Experiments were performed under specific license from the U.K. Home Office.

### Transgene induction *in vivo*

To induce the expression of the knockdown system *in vivo*, transgenic founder animals were given drinking water supplemented with Ornicure (Oropharma) for 14 days. For analysis of adult testes, pieces of testes were dissected and fixed in 4% PFA, before embedding in gelatine and cryosectioning for use in confocal analysis.

### Apple G_1_ embryo induction

Laid eggs from stock hens crossed with PB Tet-On Apple shGFP host cockerels were injected with 0.5 ml of 0.1 mg/ml doxycycline into the blunt end and sealed with tape. Eggs were incubated and embryos were dissected and imaged using a fluorescent stereomicroscope.

### Immunohistochemistry and flow cytometry analysis

Cryo-embedded tissue sections were stained with Hoechst (Sigma), mounted and imaged using either an inverted Nikon eC1 confocal microscope (Nikon) or Zeiss LSM 720 inverted confocal microscope (Zeiss). Images were analysed using Nikon Ez-C1 (Nikon) or Zen software (Zeiss) respectively. CEFS and PGCs stably transfected with PB Tet-On Apple shGFP were induced for seven days and analysed using a BD FACSAria II machine (BD Biosciences).

### Statistical analysis

All statistical analysis was performed using Minitab statistical software. A student's paired t-test was used to calculate significant difference between Apple^+^ and Apple^-^ FACS cell populations. To compare the migration efficiency of PB Tet-On AKT PGCs, a student's t-test was used to calculate the significant difference between control and dox-treated embryos.

## Results

### A single vector inducible piggyBac transposon

**Figure 1:**
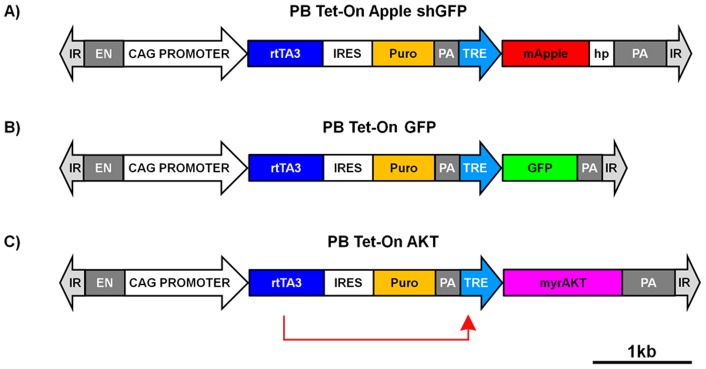
Schematic of the piggyBac ‘Tet-On’ vectors. The PB Tet-On vectors contain a CAG enhancer/promoter that drives the expression of a 3^rd^ generation reverse tetracycline transactivator (rtTA3) coupled to an IRES and puromycin resistance gene. Immediately downstream is a minimal tetracycline response element (TRE) promoter that drives: (A) Apple fluorescent protein gene containing a short hairpin (hp) RNA in the 3′ UTR against the GFP gene (B) the GFP gene (C) a constitutively active form of the human *AKT* gene. **EN** = Enhancer, **IR**  =  Inverted Repeat, **IRES**  =  Internal Ribosome Entry Site, **PA** = polyA tail, **Puro** = puromycin resistance gene.

To produce a single transposon vector containing an inducible reporter gene we used the ubiquitous promoter, CAG, a hybrid enhancer/promoter containing the CMV-IE enhancer fused to the chicken β-actin promoter and first intron [34], to drive expression of a 3^rd^ generation reverse tetracycline transactivator protein (rtTA3) [35] followed by an internal ribosome entry site (IRES) and the puromycin resistance gene (puro). This cassette provides ubiquitous expression of rtTA3, which binds to the tetracycline response element (TRE) in the presence of doxycycline (dox), and the puromycin resistance gene to allow the selection and expansion of stably transfected clones. Downstream of this cassette we cloned a minimal TRE-tight promoter which drives gene expression after binding of the rtTA3 protein. The final construct is a single selectable vector that that can be transposed into cells, allowing the conditional expression of a gene product and a shRNA targeting a gene of interest (**Fig. 1**). In this work we present three inducible vectors, one driving expression of green fluorescent protein (GFP), another driving monomeric Apple (red) fluorescent protein (Apple) expression coupled to a short hairpin RNA (shRNA) targeting the GFP gene, and a third vector driving expression of a constitutively active human *AKT* gene [26].

### Inducible gene expression and GFP knockdown in cell lines and *in ovo* electroporated chicken embryos

**Figure 2:**
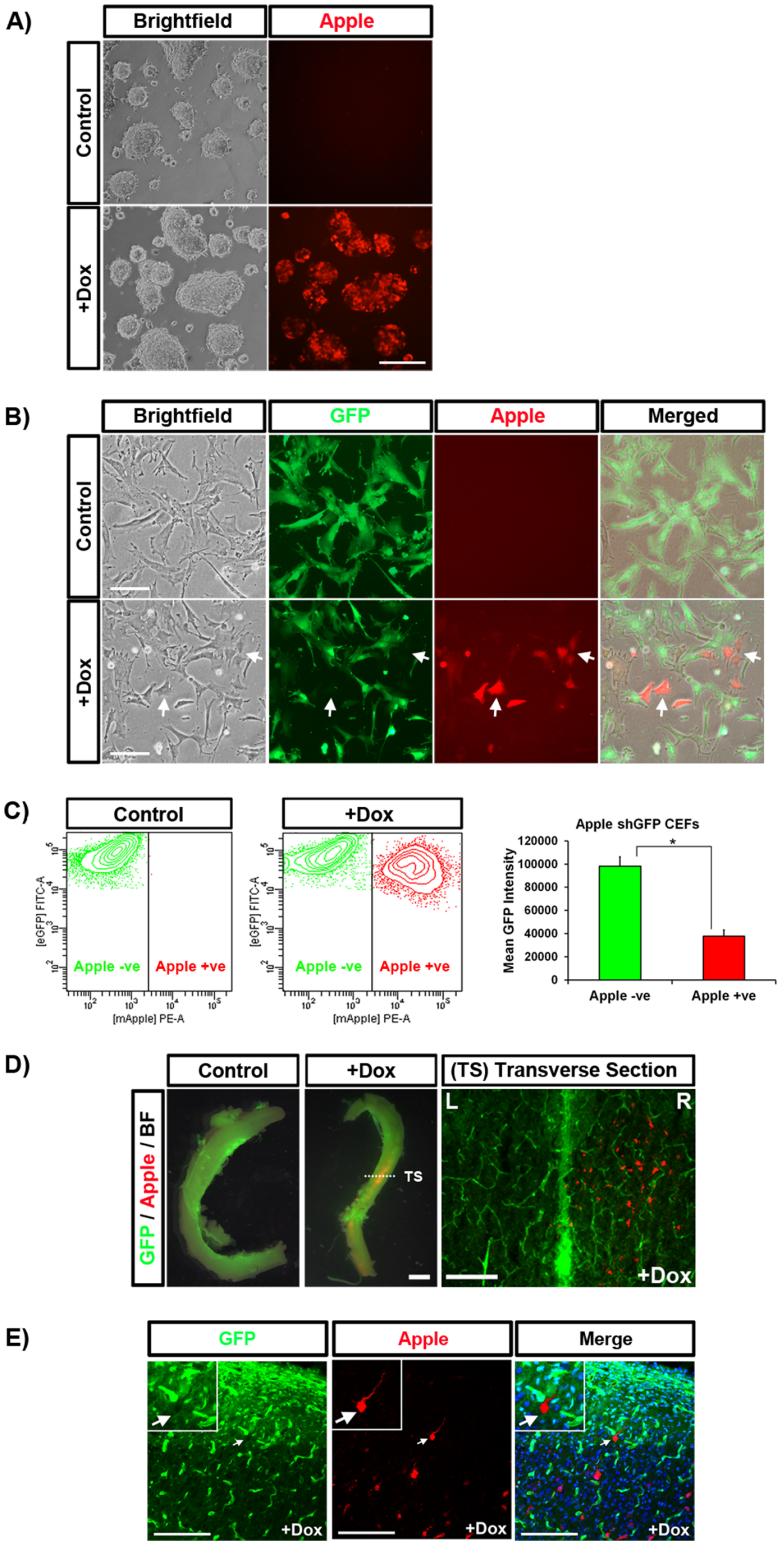
Induced transposon expression in mouse ES cells, CEFs, and embryos. (A) Mouse ES cells stably transfected with PB Tet-On Apple shGFP and treated with dox for 72 hours. (B) GFP^+^ CEFs stably transduced with PB Tet-On Apple shGFP and treated with dox for seven days. In Apple^+^ cells, GFP expression was visually reduced. (C) Flow cytometry confirmed that GFP fluorescence was significantly lower in Apple^+^ cells, * p<0.05. (D) PB Tet-On Apple shGFP was electroporated into the neural tube of stage 14HH GFP^+^ embryos. Seven days post-electroporation, the embryos were treated with dox for seven days. After dissection, Apple protein is visible in the electroporated section of the spinal cord in embryos treated with dox. (E) Confocal microscopy of a transverse section revealed Apple^+^ neurons with reduced GFP fluorescence. L  =  left, R  =  right. Scale bars: A, B = 50 µm, D middle = 1 mm, D right = 100 µm, E = 200 µm.

To demonstrate that the piggyBac vector carrying the tet-inducible cassette was functional with little background expression in the absence of dox, PB Tet-On Apple shGFP (**Fig. 1A**) was co-transfected with piggyBac transposase into mESCs. Stably transfected cells were selected with puromycin and subsequently treated with dox. Apple fluorescence was visible in induced cells (**Fig. 2A**). No expression was visible in control mESCs. We next asked if knockdown of GFP was possible from the integrated transposon. We transfected the same constructs into chicken embryonic fibroblasts (CEFs) cultured from GFP^+^ transgenic embryos (see Materials and Methods). Transfected cells were selected with puromycin and subsequently treated with dox to induce the expression of Apple and the shRNA targeting the GFP gene. One week after induction, Apple expression was visible in CEFs treated with dox (88-fold induction). Control (-dox) cells had no visible Apple expression, demonstrating the tight regulation of reporter gene expression (**Fig. 2B**). In addition, Apple positive (Apple^+^) CEFs exhibited visibly reduced GFP expression (**Fig. 2B**). FACs analysis confirmed that the population of Apple^+^ cells had a significantly reduced mean GFP expression (62%) compared with Apple^-^ cells which we used as a comparative control (**Fig. 2C**).

To demonstrate that the transposon was functional *in ovo*, we co-electroporated PB Tet-On Apple shGFP and piggyBac transposase into the neural tube of stage 14 HH embryos. Embryos were incubated for one week to allow for the degradation of non-integrated plasmid then induced with dox. Similar to our *in vitro* observations, Apple fluorescence was only detected in the spinal cords of embryos treated with dox (**Fig. 2D**). Transverse sections of the spinal cords taken from dox-treated embryos identified Apple^+^ neurons in the electroporated side of the spinal cord (**Fig. 2D**). When visualised at higher magnification it was apparent that some of the Apple^+^ neurons also exhibited reduced GFP expression in comparison to neighbouring neurons (**Fig. 2E**). These results confirm that expression from the transposon is tightly regulated in cell culture and *in ovo*.

### GFP knockdown in cultured primordial germ cells

**Figure 3:**
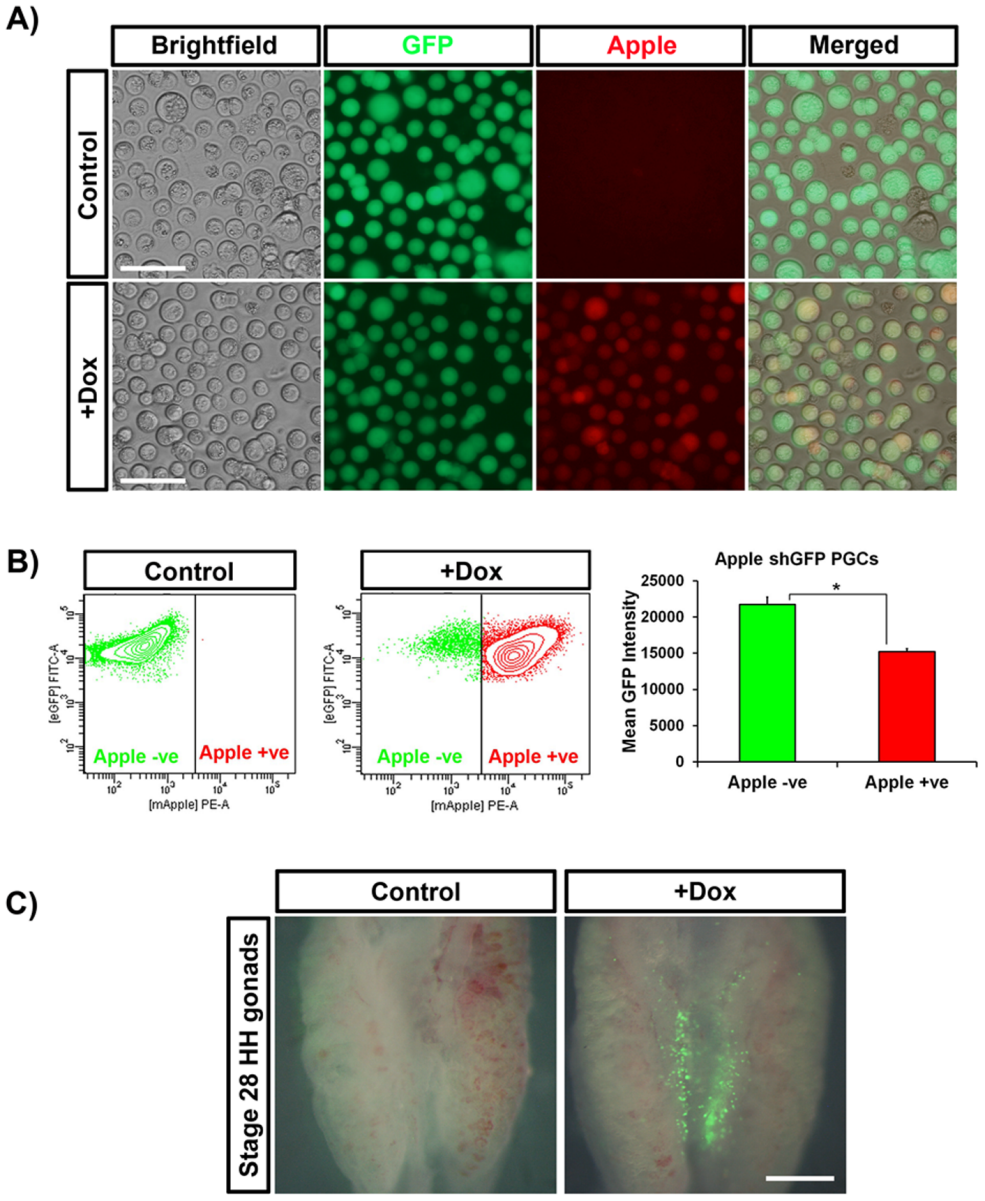
Reporter gene induction in PGCs *in vitro* and *in ovo*. (A) GFP^+^ PGCs stably transfected with PB Tet-On TRE Apple shGFP were treated with dox for seven days. (B) GFP expression was significantly lower in Apple^+^ cells by flow cytometry, * p<0.05. PGCs stably transfected with PB Tet-On TRE GFP were injected into donor HH16 embryos. (C) GFP expression was visible in the gonads of dox-treated embryos 72 hrs post injection (stage 28 HH). Scale bars: A = 50 µm, B = 1 mm.

We next demonstrated that the inducible vectors could be used to modify the germ cell lineage. Cultured chicken PGCs were co-transfected with PB Tet-On Apple shGFP and with piggyBac transposase and selected with puromycin. PGCs were treated with dox for seven days and observed for fluorescence. We found that Apple expression was not detectable in control cells but highly induced (40-fold) after treatment with dox (**Fig. 3A**). Flow cytometry revealed a significant reduction in GFP expression (32%) in Apple^+^ PGCs, indicating that this system can be used to knockdown gene expression in PGCs (**Fig. 3B**). Although PGCs exhibited high levels of Apple reporter expression, PGCs showed a markedly weaker knockdown of GFP expression (62% versus 32%) indicating that knockdown efficiency varies between cell types.

### Induction of gene expression in the germ cell lineage *in ovo*

Cultured chicken PGCs co-transfected with PB CAG Tet-On GFP, a Tet-On vector that conditionally expresses GFP, and with piggyBac transposase. PGCs were selected with puromycin and proliferated *in vitro*. These PGCs were then injected into the developing vasculature of stage 16HH embryos. Dox was concurrently injected into the yolk directly underneath the embryo. After 72 hrs incubation, during which period the endogenous germ cells migrate to and colonise the developing gonads, we examined the embryos for GFP expression. We observed that in stage 28HH embryos treated with dox, GFP^+^ cells were present in the gonads, validating the vector for inducible transgene expression in PGCs *in ovo.* (**Fig. 3C**).

**Figure 4:**
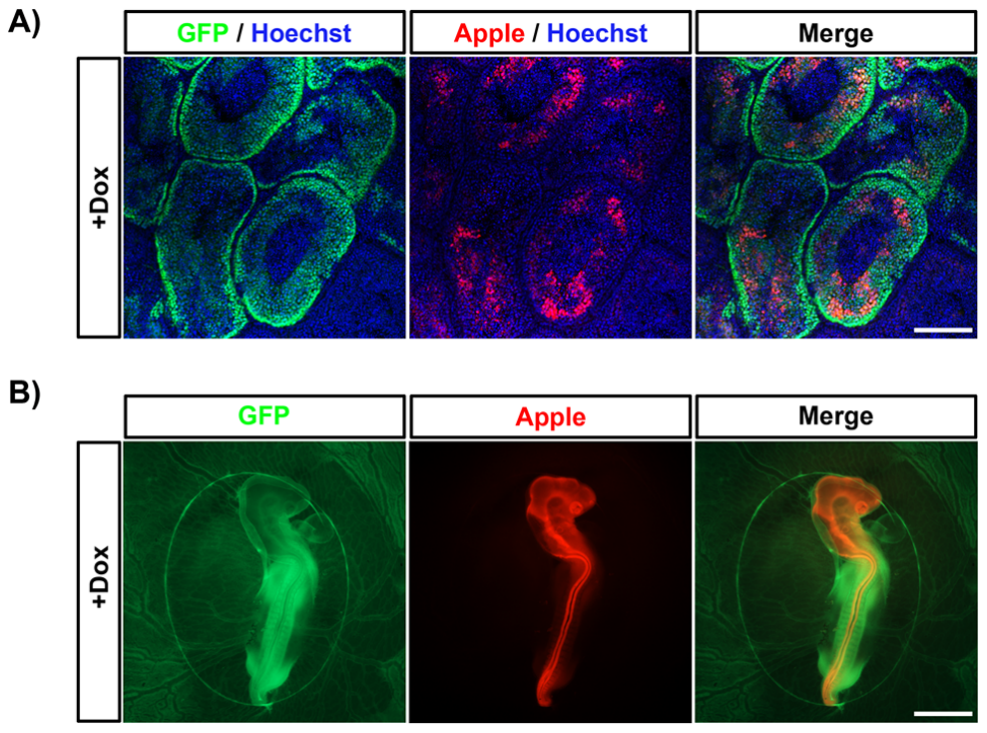
Reporter gene induction in spermatogonial stem cells of adult roosters *in vivo* and in G_1_ embryos. PGCs stably transfected with PB Tet-On TRE Apple shGFP were injected into donor stage16HH embryos, hatched and raised to sexual maturity. After two weeks of treatment with doxycycline the rooster was killed and sections of the testes were imaged for (A) GFP and Apple fluorescence (B). The host roosters were mated to wildtype hens and newly-laid eggs were injected with dox and imaged for GFP and Apple fluorescence at day three of incubation. Scale bars: A = 100 μm, B = 2 mm.

In similar experiments, we injected cultured male GFP^+^ chicken PGCs stably transfected with PB Tet-On Apple shGFP into the bloodstream of stage 16HH recipient embryos. Injected embryos were hatched and the males were raised to sexual maturity. The roosters were treated with dox (see Materials and Methods) for two weeks, euthanized, and the testes were examined for fluorescence. We observed large domains of GFP^+^ cells lining the seminiferous tubules of the testes, extending from the abluminal surface to the forming spermatids at the centre of the tubules (**Fig. 4A**). Apple fluorescence was visible in a similar domain (**Fig. 4A**), indicating that transgene expression was induced in cells undergoing gametogenesis in the adult bird.

To generate transgenic offspring containing the inducible-transgene transposon vector, derived from transposon-modified GFP^+^ PGCs, we mated similar host roosters with wildtype hens and screened their offspring for the presence of the vector (**Table S1**). Several GFP^+^ G_1_ chicks were produced, indicating that the injected PGCs could form functional sperm. Surprisingly, no transgenic chicks carrying the integrated vector were hatched. We analysed G_1_ embryos for integration of the transposon by PCR and Southern blot analysis. We found that all embryos carrying the vector died, by day six of incubation (**Table S1 and data not shown**). Transgenic embryos that developed during the first few days of incubation were analysed and displayed morphological defects in the extra-embryonic vascular system at day three of incubation (**Fig 4B** **and Table S1**). Southern blot analysis of six transposon-positive embryos, to identify junctional fragments between the integrated vector and genomic DNA at the integration site, indicated that the embryos each contained a single copy of the vector, each at independent genomic locations (**Fig. S1**). Thus, the observed embryonic death was not due to the specific integration site of the vector.

To demonstrate that the transgene was inducible in G_1_ embryos, fertile G_1_ eggs were treated with dox and incubated for three days. All transgenic embryos expressed the Apple reporter gene throughout the early embryo (n = 10) (**Fig 4B**). We conclude from these experiments that the integrated vector is functional in G_1_ embryos and that ubiquitous expression of the TA protein is likely lethal to early chick embryos.

### Constitutive AKT signalling disrupts primordial germ cell migration

**Figure 5:**
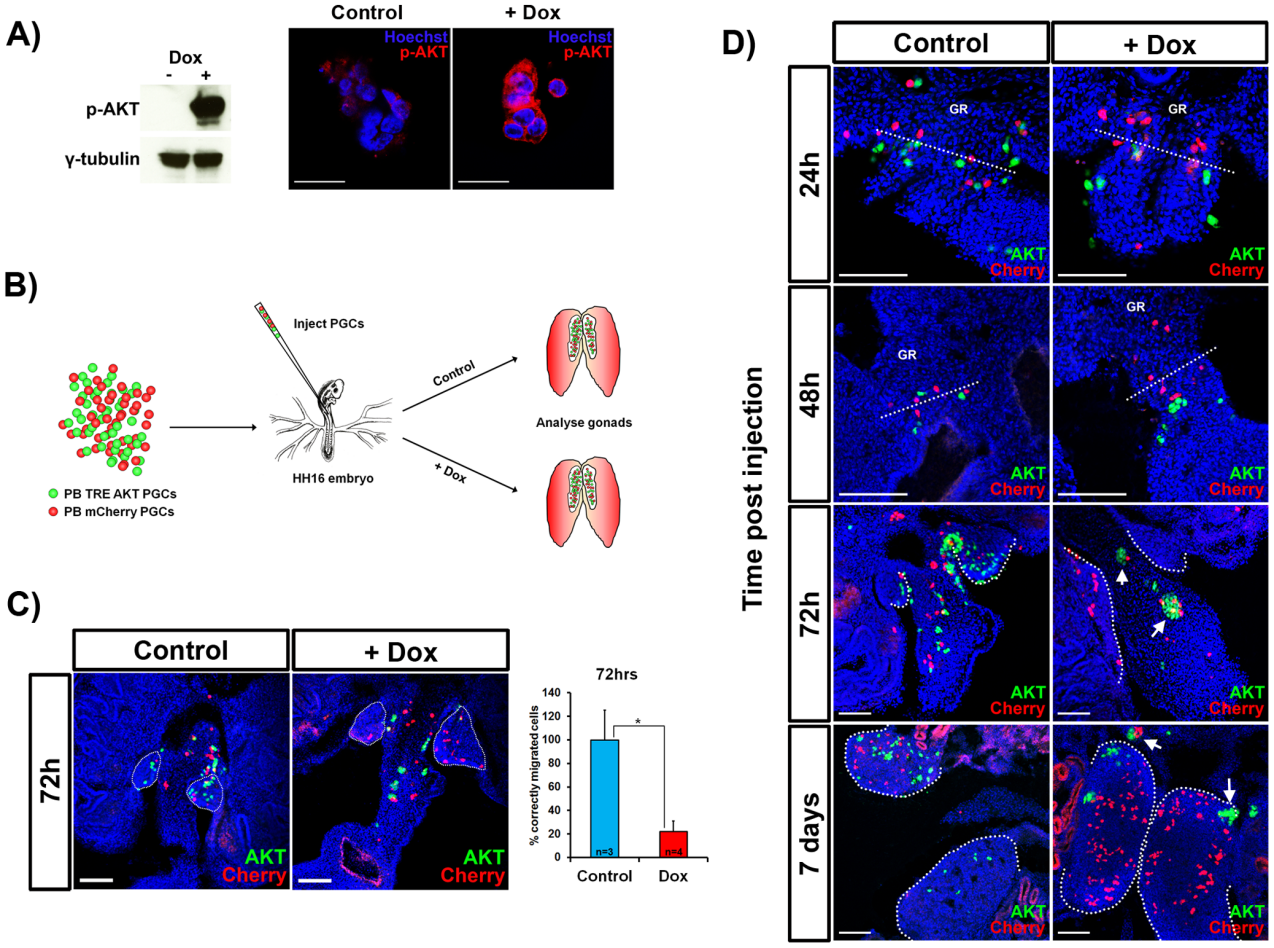
Expression of a constitutively active AKT protein inhibits PGC migration and leads to extra-gonadal clustering of PGCs. (A) Western blot and immunofluorescence analysis of PB Tet-On AKT-transfected:GFP^+^ PGCs treated with dox for 72 hours and assayed with an antibody to phosphorylated AKT. (B) Schematic representation of the migration assay to analyse the effect of AKT activation. Control PGCs expressing mCherry (red) were mixed with equal numbers of uninduced GFP^+^ PB Tet-On AKT PGCs (green) and injected into stage 16HH embryos and treated with dox. (C) After 72 hrs incubation, the majority of PGCs overexpressing AKT (+dox) failed to migrate to the gonad. (D) Constitutive AKT activation retards PGC migration at early time points and prevents PGCs from colonising the gonads at later stages, resulting in formation of large clusters of PGCs extra-gonadally (arrows). Dashed lines indicate the average front of the injected cherry PGCs after 24 h/48 h and at later stages highlight the developing gonads. Dorsal: top of page. Scale bars: A = 50 µm, C & D = 100 µm. *p<0.05.

PGC migration depends on correct directional mobilisation of the cytoskeleton and interactions with the surrounding extracellular substrate [36], [37]. The serine/threonine protein kinase AKT has been shown to play a pivotal role in controlling aspects of cell migration. To investigate the role of AKT in early chicken germ cell migration we co-transfected PGCs with PB Tet-On AKT, a Tet-On vector that drives a constitutively active, myristolated form of human *AKT* and piggyBac transposase [26]. Human and chicken AKT have 98% conservation of amino acids (data not shown), so conservation of function is predicted. The transfected cells were selected with puromycin, induced with dox and assayed by western blot analysis and immunofluorescence (**Fig. 5A**). A strong band of the correct molecular weight was detected in the dox-treated PGCs and immunofluorescence reactivity at the plasma membrane. To investigate whether activation of the AKT pathway has an effect on cell migration we designed a PGC migration assay (**Fig. 5B**). Equal numbers of AKT stably-transfected GFP^+^ PGCs and control PGCs, transfected with a PB vector for constitutive expression of Cherry fluorescent protein, were injected into stage 16HH embryos. Embryos were treated with dox to induce expression of constitutively-active AKT, and the locations of GFP^+^ and Cherry^+^ cells measured after 72 hours, to assess migration efficiency. GFP^+^ cells were significantly delayed in migration to the gonad in comparison to Cherry^+^ cells (**Fig. 5C**). We next assayed migration at earlier and later timepoints. GFP^+^ cells were visibly retarded in migration compared to Cherry^+^ cells after both 24 and 48 hours (**Fig. 5D**). Notably, one week after injection the PGCs had formed large clusters in dox injected embryos and did not reach the gonad (**Fig. 5D**). These results demonstrate that AKT signalling can effect PGC migration to the forming gonad.

## Discussion

Transposable vectors systems that can conditionally alter gene expression *in vivo* are a valuable tool for research in many biological areas including induced pluripotency, immune response, cancer cell growth and cell migration [17]–[21]. Single vector inducible transgene transposons offer advantages over existing transposon systems currently used in the chicken embryo [38], [39], as only a single vector integration is required to deliver the inducible transgene system. Conditional expression systems are also ideal for regulated expression of proteins that can be toxic to cells after chronic expression, such as the AKT molecule used in this study.

Transgenic chickens have proven to be invaluable models for biological, agricultural, and medical research [40]–[42]. The use of cultured PGCs and transgenesis using transposon vectors, as a route to production of transgenic chickens, is a valuable tool for such applications [16], [22], [43], [44]. The single vector transposon used here is a novel advance over previous inducible transposon vectors and the generation of conditional knockdown birds will be useful for the study of disease resistance and developmental processes. This approach could be applied to mammalian and zebrafish models. The presence of the integrated vector unexpectedly proved toxic to early chicken embryos. This toxicity is not due to either the integration site or integration of multiple copies of the vector so we deduce that it is a result of ubiquitous expression of the TA protein in early embryos. This result was not predicted since a similar construct driven by the CAG promoter in mice had no adverse effects on development [4]. However, CAG driving TA protein has been noted to be toxic in other mouse transgenic models [45], [46]. This suggests that constructs used in mouse transgenesis cannot be simply transferred to avian models. A possible solution will be to replace the ubiquitous CAG promoter in the vector with lineage-specific promoters to limit the expression domain of the TA protein.

PGC migration in vertebrates involves recognition of a chemoattractant gradient and active cell migration [36], [37]. Signalling of the chemokine SDF-1 through the CXCR4 receptor leads to directed changes in cell motility and cell adhesion to neighbouring germ cells and the extracellular matrix [47], [48]. This is accompanied by directed remodelling of the actin cytoskeleton, with membrane blebbing and pseudopodia extension [49]. The growth factor, stem cell factor (SCF), mediates PGC survival and was also shown to be a chemokine involved in mouse PGC migration [50]. SCF binds to the c-kit receptor and signals through PI3Kinase to phosphorylate many downstream effectors, key amongst these being serine/threonine kinase AKT. In mouse, SCF has been shown to be important for the continued motility of PGCs during migration but not their direction of migration [51]. In mice mutant for PTEN, an inhibitor of PI3Kinase and AKT signalling, migratory germ cells showed an increase in AKT phosphorylation and many germ cells were delayed along their normal pathway of migration [52].

In zebrafish, signalling through PI3Kinase is also required for maintaining the mobility and speed of migration but not the direction of PGC migration [53]. In these cells, AKT is not localised to the leading edge of the migrating cells, indicating that it is not involved in the direction of migration. In chicken PGCs, inhibition of PI3Kinase using LY400032 leads to cessation of proliferation in cultured chicken PGCs, but its role in germ cell migration has not been investigated [23], [54]. Our current data suggests that the level of AKT signalling may be important for maintaining PGC migration but not the direction of migration.

## Supporting Information

Figure S1 **Southern blot analysis of G1 embryos from PB Tet-On Apple shGFP transduced PGCs.** Genomic DNA samples were digested with *Mfe*I and hybridized with a probe for TA. Analysis of six G_1_ embryos revealed single independent transposon integrations.
(TIF)

Table S1 **PCR analysis of hatched chicks and embryos.** Genomic DNA from hatched chicks and developed day 18 embryos was analysed by PCR for the presence of the PB Tet-On Apple shGFP transgene and the GFP transgene.
(DOCX)
