# Supplementary figures and images for: A Novel Piggybac Transposon Inducible Expression System Identifies a Role for Akt Signalling in Primordial Germ Cell Migration
Source: PLoS One. 2013 Nov 4;8(11):e77222. doi: 10.1371/journal.pone.0077222 (PMC3817190; doi:10.1371/journal.pone.0077222)

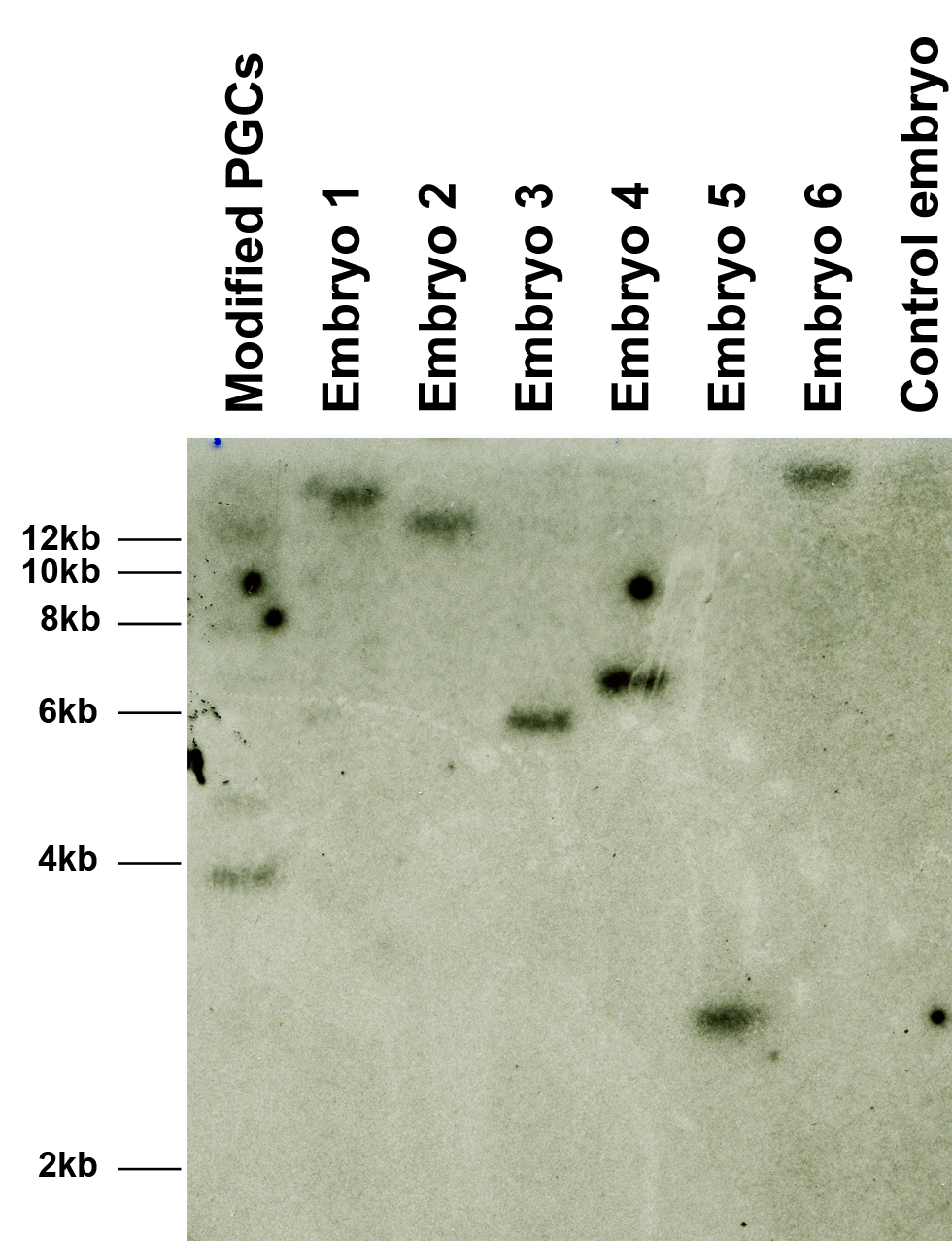

Supplement: Figure S1 — Southern blot analysis of G1 embryos from PB Tet-On Apple shGFP transduced PGCs. Genomic DNA samples were digested with MfeI and hybridized with a probe for TA. Analysis of six G1 embryos revealed single independent transposon integrations. (TIF) [file pone.0077222.s001.tif]
