# Supplementary material for: A Novel Piggybac Transposon Inducible Expression System Identifies a Role for Akt Signalling in Primordial Germ Cell Migration
Source: PLoS One. 2013 Nov 4;8(11):e77222. doi: 10.1371/journal.pone.0077222 (PMC3817190; doi:10.1371/journal.pone.0077222)
Supplement: Table S1 — PCR analysis of hatched chicks and embryos. Genomic DNA from hatched chicks and developed day 18 embryos was analysed by PCR for the presence of the PB Tet-On Apple shGFP transgene and the GFP transgene. (DOCX) [file pone.0077222.s002.docx]

| **Founder male** |  | **Eggs** | | **Offspring ( transmission )** | | |
| --- | --- | --- | --- | --- | --- | --- |
| **Bird #** | **Semen PCR** | **Set** | **Hatched** | **GFP+** | **Apple+** | **GFP+ / Apple+** |
| App #3-2 | 30% | 196 | 83 (42%) | 9 (11%) | 0 | 0 |
| App #3-4 | 10% | 172 | 98 (57%) | 3 (3%) | 0 | 0 |
|  |  |  |  |  |  |  |
|  |  |  |  |  |  |  |
|  |  |  |  |  |  |  |
| **Founder male** | **Incubated eggs** | | **Analysis of developed embryos** | | | |
| **Bird #** | **Candled** | **Developed (%)** | **Negative** | **GFP+** | **Apple+** | **GFP+ / Apple+** |
| App #3-2 | 73 | 25 (34%) | 4 (16%) | 0 (0%) | 12(48%) | 9 (36%) |
| App #3-4 | 18 | 4 (22%) | 0 (0%) | 1 (25%) | 2 (50%) | 1 (25%) |
